# Complete Genome Sequence, Molecular Characterization and Phylogenetic Relationships of a Temminck’s Stint Calicivirus: Evidence for a New Genus within *Caliciviridae* Family

**DOI:** 10.3390/microorganisms10081540

**Published:** 2022-07-29

**Authors:** Alina Matsvay, Marina Dyachkova, Anna Sai, Valentina Burskaia, Ilya Artyushin, German Shipulin

**Affiliations:** 1 Federal State Budgetary Institution “Centre for Strategic Planning and Management of Biomedical Health Risks” of the Federal Medical Biological Agency, 119121 Moscow, Russia; mdyachkova@cspmz.ru (M.D.); asay@cspmz.ru (A.S.); shipgerman@gmail.com (G.S.); 2 Center of Life Sciences, Skolkovo Institute of Science and Technology, 143026 Moscow, Russia; valya.burskaya@gmail.com; 3 Faculty of Biology, Lomonosov Moscow State University, 119991 Moscow, Russia; sometyx@gmail.com

**Keywords:** *Caliciviridae*, *Sanovirus*, Temminck’s stint calicivirus, genome annotation, phylogenetics

## Abstract

*Caliciviridae* is a family of viral pathogens that naturally infects vertebrates, including humans, and causes a range of highly contagious infectious diseases. Caliciviruses are not well studied because of the lack of a universal approach to their cultivation; however, the development of molecular genetics and bioinformatics methods can shed light on their genetic architecture and evolutionary relationships. Here, we present and characterize the complete genome sequence of calicivirus isolated from a sandpiper—Temminck’s stint (*Calidris temminckii*), preliminarily named Temminck’s stint calicivirus (TsCV). Its genome is a linear, non-segmented, single-stranded (+sense) RNA with genome organization typical of avian caliciviruses. Comparative studies have shown significant divergence of the nucleotide sequence of the TsCV genome, as well as the amino acid sequence of the major capsid protein from all publicly available genomic and protein sequences, with the highest genome sequence similarity to unclassified *Ruddy turnstone calicivirus A* (43.68%) and the lowest pairwise divergence of the major capsid protein with unclassified goose calicivirus (57.44%). Phylogenetic analysis, as well as a comparative analysis of the homologous proteins, showed evidence of another separate genus within the *Caliciviridae* family—previously proposed, but not yet accepted by International Committee on Taxonomy of Viruses (ICTV)—the *Sanovirus* genus, which combines seven previously unclassified genomic sequences of avian caliciviruses, including the newly discovered TsCV, which we propose to consider as a separate species.

## 1. Introduction

Caliciviruses are small non-enveloped pathogens with a single-stranded RNA genome varying from 6.4 to 8.5 kb in length belonging to the *Caliciviridae* family [1]. These viruses are known to infect mammals, birds and fish [2,3]. According to the current International Committee on Taxonomy of Viruses (ICTV) report, 11 genera are currently accepted within the *Caliciviridae* family—*Lagovirus, Norovirus, Nebovirus, Recovirus, Sapovirus, Valovirus, Vesivirus, Bavovirus, Nacovirus, Minovirus and Salovirus* [4], with each genus including one to two species [5]. The taxonomic classification of *Caliciviridae* is based on the protein sequence of the major capsid protein (VP1) with isolates with less than 60% sequence identity being assigned to different genera [4]. However, a number of calicivirus isolates currently remain unclassified and several new genera are proposed, including *Sanovirus* [6] and *Secalivirus* [7].

The genomic RNA of caliciviruses is organized in one of two ways. In the genomes of representatives of *Norovirus*, *Recovirus* and *Vesivirus* genera, three open reading frames (ORFs) are present. ORF1 of murine norovirus encodes a polyprotein, which is cleaved into six to seven nonstructural proteins (NS): N-term (NS1/2), NTPase (helicase, NS3), 3A-like (NS4), VPg (virion genome-linked protein, NS5), viral protease (NS6) and RNA-dependent RNA polymerase (RdRp, NS7) [8]. Similarities with homologous proteins of other positive-sense single-stranded RNA viruses were used to reveal the functions of some nonstructural proteins of caliciviruses (NTPase, VPg, protease and RdRp) [9]. The calicivirus NTPase participates in viral replication, unwinds dsRNA intermediates, remodels structured RNA and forms vesicular structures for replication [10,11]; the caliciviral VPg is used as primer for the replication of a viral genome in host cells [12]; proteolytic cleavage of the viral polyprotein is performed by the calicivirus protease [13]; and the RdRp replicates the viral genome [14]. A major capsid protein (VP1) is encoded by ORF2 and ORF3 encodes a minor capsid protein (VP2). In murine norovirus, an additional ORF, ORF4, was detected encoding virulence factor 1 (VF1), lying within ORF2 with 1 nucleotide shift [15]. In genera *Lagovirus, Nacovirus, Nebovirus, Sapovirus* and *Valovirus*, on the other hand, only two ORFs are present, with VP1 and the nonstructural polyprotein being encoded together by ORF1 and VP2 being encoded by ORF2. The 3′ end of the RNA genome is polyadenylated and the 5′ end is linked to VPg [9]. 

Cup-shaped depressions located on the capsid surface of caliciviruses are considered to be a unique morphological feature of the group [16]. The capsid of a calicivirus consists of 180 copies of VP1 in three different conformers (A, B and C). A short N-terminal arm, a shell domain and a protruding domain form a mature capsid protein. VP2 is also integrated into the virion, but the copy number is comparatively lower [17].

Birds are known hosts for caliciviruses from genera *Bavovirus*, *Nacovirus* and *Norovirus*. Bird-infecting caliciviruses are pathogens of known importance, since they are able to infect poultry, including chicken [18], turkey [19] and geese [20]. Additionally, birds are already known to be carriers for pathogens that are able to infect humans, such as West Nile virus (WNV) [21,22,23], Japanese encephalitis virus [24] and several subtypes of avian influenza virus [25,26,27]. At least some caliciviruses are suggested to be able to cross the species barrier [28,29], which makes migrating birds a reservoir with potential epidemiological importance. In this study, we have sequenced, assembled and characterized a complete genome of previously undescribed Temminck’s stint calicivirus (TsCV) isolated from Temminck’s stint. The amino acid sequence of the TsCV major capsid protein, which is used for taxonomic classification of the *Caliciviridae* family, is more than 60% diverged from any other classified caliciviruses, suggesting that the newly identified virus does not belong to any of the ICTV-accepted genera, and shows the highest similarity (57.4%) to currently unclassified goose calicivirus (NCBI accession number KY399947.1), which was previously described as a founding member of the proposed *Sanovirus* genus [6]. 

## 2. Materials and Methods

### 2.1. Sampling 

The sample under the study was collected in 2017 on the banks of the Yenisei River in Krasnoyarsk Region (Russia, Siberia) [30] and belongs to the Temminck’s stint (*Calidris temminckii*), a small-sized shorebird of the Sandpipers family (*Scolopacidae*). The biological sample was collected without direct contact with animals; no invasive interventions on animals were carried out. The birds have been observed and identified by qualified zoologists at close distance from a camouflaged hideout. Bird droppings were collected immediately after discharge, taking only the surface part of the fecal pellet to avoid contamination. 

To ensure the preservation of the nucleic acids of viral pathogens, fecal samples were placed in sterile tubes containing the transport medium (reagent for transportation and storage of clinical material, Amplisens, Moscow, Russia). After transportation to the laboratory, the sample was stored in a low-temperature refrigerator.

### 2.2. Sample Preparation and Sequencing

For the extraction of nucleic acids, an Allprep DNA/RNA mini kit (Qiagen, Hilden, Germany) was used. All manipulations were carried out in accordance with the manufacturer’s instructions. Preliminary screening for avian viral pathogens was carried out as described earlier [30].

Library preparation for high-throughput sequencing was performed with a NEBNext Ultra II RNA Library Prep Kit (New England Biolabs, Ipswich, MA, USA) following the manufacturer’s recommendations for partially degraded samples. No additional steps involving depletion and any kind of enrichment were used. Sequencing with MiSeq Reagent Kit v2 (500-cycles) (Illumina, San Diego, CA, USA) on the Illumina MiSeq platform (Illumina, San Diego, CA, USA) resulted in 1.276 M paired-end reads per the sample under the study.

### 2.3. Assembly and Genome Annotation

SPAdes software v.3.15.3 [31] (CAB SPbU, St. Petersburg, Russia) was used for de novo metagenomic assembly. The main script with standard parameters was used, except for the activation of the “careful” option. The resulting contigs were used for taxonomic classification by nucleotide and translated protein sequences using the BLAST algorithm [32] with Nr/Nt and the NCBI Taxonomy databases [33]. The taxonomic classification of the host was further confirmed by the analysis of contigs related to eukaryotes (Table A1). Contigs attributed to the *Caliciviridae* family were used to obtain draft whole-genome assembly using the SeqMan NGen program (DNASTAR, Madison, WI, USA).

Additionally, the original raw reads were mapped to the draft whole-genome assembly to perform errors correction. BWA v.0.7.17 [34] was used for used for mapping and Samtools package v.1.10 [35] for operations with sam/bam files. Assembly check and correction was performed using Tablet program v.1.19.09.03 [36]. The quality and integrity of the 3′ end of the assembly was assessed manually.

An NCBI open reading frame finder [37] was used to annotate the open reading frames (ORFs). The following search parameters were used: minimal ORF length 150 amino acids, genetic code 1 and start codon “ATG only”. Protein-coding genes were identified by analyzing homologous protein sequences using the BLAST algorithm [32] with translated nucleotide search. The domain enhanced look-up time accelerated BLAST (DELTA-BLAST) algorithm [38] was used to detect highly distant protein homologues in the absence of significant hits in the standard blast search. Visualization of the annotated sequence and search for characteristic conserved protein motifs were carried out using the SnapGen Viewer software [39] (Dotmatics, Boston, MA, USA).

### 2.4. Comparative Analysis

The annotated genome assemblies used in the comparative analyses were retrieved from the GenBank database [40] [date of access: 15 May 2022].

The pairwise alignments of whole-genome sequences, and amino acid sequences of annotated major capsid proteins of representative genomes of each *Caliciviridae* genus (Table A2), unclassified *Caliciviridae* representatives (Table A3) and TsCV were constructed using MAFFT software [41] for every possible pair of genomes and protein sequences. Pairwise identity for each nucleotide alignment was calculated using DistanceCalculator from Bio.Phylo.TreeConstruction module of BioPython [42] and ‘identity’ model for calculation of nucleotide divergence. Evolutionary divergence between VP1 amino acid sequences was estimated using MEGA11 software [43] with a frequencies model with a gamma distribution of variation including invariant sites, as described in [4].

### 2.5. Phylogenetic Analysis

To build a phylogenetic tree of representatives of the *Caliciviridae* family, including TsCV, we used the amino acid sequences of annotated major capsid proteins of the above set of genomes (Table A2 and Table A3). The multiple sequences alignment was performed using MAFFT [41]. The maximum likelihood unrooted tree was generated using RAxML-NG v.1.0.2 according to the recommendations of the ICTV [4] with the only improvement being the choice of an evolutionary model, which was determined using PartitionFinder v.2.1.1 [44] under the corrected Akaike (AICc) and the Bayesian (BIC) information criteria. LG + I + G + F was determined as the best-fitting model. Partial and duplicate sequences (YP_009666353.1, AFH89835.1, YP_009028574.1, AAB60927.1, QXO14962.1, UNY48346.1 and QXO14970.1) have been removed from further analysis. Bootstrapping converged after 650 replicates.

To build a high-resolution phylogenetic tree of TsCV and its closest relatives, we used the nucleotide sequences of the complete genomes of TsCV and a set of the most closely related members of the *Caliciviridae* family according to the criterion of percent sequence identity (Table A3). This list includes representatives of the genera *Bavovirus* and *Nacovirus*, as well as unclassified caliciviruses. The multiple sequences alignment was performed using MAFFT [45]. To eliminate poorly aligned and diverged regions, Gblocks v.0.91b [46] was used with the default parameters. The analysis of possible recombination events was performed using the GARD program implemented in the HyPhy software v.2.5.40 [47]. The maximum likelihood unrooted tree was generated using RAxML-NG v.1.0.2 [48] with GTR + I + G as the most parameter-rich model [49,50]. Bootstrapping converged after 300 replicates. The trees were visualized and rooted in midpoint using iTOL v.6 [51].

### 2.6. Species Demarcation

We used three approaches that propose de novo species partitions to confirm the species status of the TsCV virus as described previously in [52]: the GMYC [53], bPTP [54] and ASAP [55] methods. Calculations have been carried out for the ICTV set of representative genomes of the *Caliciviridae* family (Table A2), supplemented with genome sequences of unclassified caliciviruses that are most closely related to TsCV (Table A3). We used the single-threshold version of the GMYC method, since the multiple-threshold version tends to overestimate the number of species partitions [53]. The ultrametric timetree as input tree for GMYC analysis was obtained by applying the RelTime method [56,57] implemented in the MEGA-11 software v.11.0.11 [58] using the appropriate evolutionary model. bPTP analysis was run with default parameters using 500,000 MCMC generations. A matrix of patristic distances as input matrix for ASAP analysis was obtained using the cophenetic.phylo function implemented in the R package ape [59].

### 2.7. Protein 3D Structure Prediction

To predict 3D structures of TsCV proteins we used a machine learning approach, AlphaFold2, which is able to predict protein structures with an accuracy close to experimental [60]. To build a multiple alignment, we searched for homologues in the following databases: Uniref90 [61], Mgnify [62], BFD [63], UniClust30 [64] and pdb70 [65]. We obtained five relaxed models and five unrelaxed models for each protein ranging by per-residue confidence score (pLDDT). For each protein, the model with the best pLDDT score was chosen for subsequent analysis. Visualization for all individual proteins and structures was performed using UCSF Chimera [66]. Comparison of 3D structures was carried out using the “match maker” function of the UCSF Chimera [66]. 

## 3. Results

### 3.1. Annotation of TsCV Genome and Comparative Analysis

**Figure 1:**
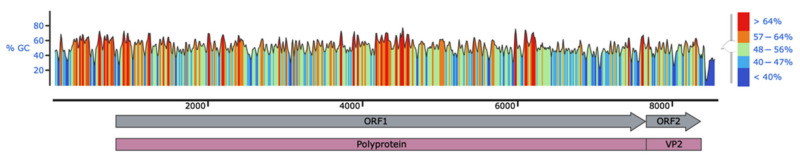
Schematic view of Temminck’s stint calicivirus (TsCV) genome. Open reading frames (ORFs) are indicated as gray arrows indicating the direction of transcription; the regions of the genes encoding the indicated protein sequences are marked in pink. A color scale from blue (minimum value) to red (maximum value) indicates G + C content.

The genome of Temminck’s stint calicivirus (TsCV) is a linear, non-segmented, single-stranded positive-sense RNA, comprised of 8575 bases with an average G + C content of 51.73% (Figure 1). 

The TsCV genome showed significant divergence from all publicly available genomic sequences with the highest similarity to unclassified *Ruddy turnstone calicivirus A* (MH453861.1, 43.68%) [67] (Table 1). The G + C proportion is also most similar to the nucleotide composition of the *Ruddy turnstone calicivirus A* genome. Open reading frames (ORFs) prediction showed genome structure typical of avian caliciviruses—TsCV coding regions are organized into two ORFs: ORF1 of 6849 bases and short ORF2 of 711 bases separated by 1 nucleotide frameshift (Figure 1). However, the relative position of open reading frames differs from its closest relatives—in the TsCv genome, the first and second open reading frames do not overlap, while for the genomes of its closest relatives the overlap is from 18 to 74 nucleotides, according to their annotation (Table 1). In addition, the length of the nucleotide sequence of the ORF1 is noticeably shorter, whereas the non-transcribed regions and the ORF2 sequence show the average lengths.

The ORF1 translation product was identified as polyprotein by analysis of homologous sequences. The protein sequence encoded by ORF2 was identified as VP2 protein using the DELTA-BLAST algorithm and the NCBI’s Conserved Domain Database (the only match was VP2 protein of grey teal calicivirus, QDY92333.1 [68]). The calculated molecular weight of the polyprotein and VP2 were 249.5 and 25.5 kDa, respectively. 

**Figure 2:**
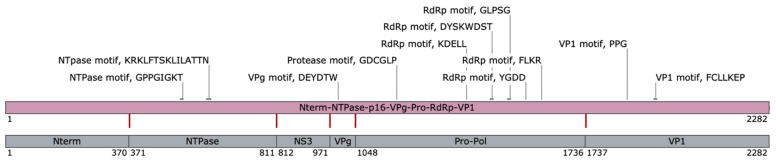
Schematic view of predicted cleavage map of TsCV polyprotein. Red lines indicated predicted cleavage sites; regions of mature proteins are delimited by gray rectangles; callouts list identified protein motifs that are conserved for caliciviruses.

ORF1 encoded an immature polyprotein of 2282 aa, which contained characteristic protein motifs conserved in caliciviruses: NTpase/helicase motifs ^526^GPPGIGKT^533^ and ^603^KRKLFTSKLILATTN^617^; VPg motif ^992^DEYDTW^997^; protease motif ^1169^GDCGLP^1174^; RdRp motifs ^1379^KDELL1^383^, ^1453^DYSKWDST^1460^, ^1556^YGDD^1559^ and ^1603^FLKR^1606^; and VP1 (major capsid protein) motifs ^1859^PPG^1861^ and ^1944^FCLLKEP^1950^ (Figure 2).

The prediction of cleavage sites was based on the alignment of the amino acid sequence of the polyprotein of TsCV and goose calicivirus (KY399947, [6])—the one fully annotated sequence of the closest relatives to date, caliciviral 3C-like protease cleavage sites preferences [69] and average weights of mature proteins. The cleavage sites of the polyprotein were predicted to be: E^370^/G, Q^811^/N, Q^971^/G, E^1047^/G and E^1736^/S. Based on the indicated cleavage sites, the molecular weights of mature proteins were predicted to be 40.7 kDa for Nterm protein (370 aa), 48.5 kDa for NTPase (441 aa), 18 kDa for NS3 protein (160 aa), 8.3 kDa for VPg protein (76 aa), 75.8 kDa for Pro-Pol (689 aa) and 58.2 kDa for major capsid protein (546 aa) (Figure 2).

### 3.2. Taxonomic Classification of TsCV by ICTV Criteria

According to the International Committee on Taxonomy of Viruses (ICTV), in the *Caliciviridae* family, the amino acid sequence of the major capsid protein (VP1) is used for taxonomic classification wherein the criterion for species demarcation is the divergence of the VP1 amino acid sequence of more than 60% [4]. 

We calculated the divergence of the VP1 amino acid sequence of TsCV and the VP1 sequences of representatives of each of the accepted genera. A set of representative sequences provided by ICTV was used for calculations (Table A2). Thus, according to the accepted criterion, TsCV cannot be assigned to any of the accepted genera of the *Caliciviridae* family, since all the given divergence values, with their standard deviations considered, are more than 60% (Table 2). The VP1 sequence of the virus is closest to the representatives of the *Nacovirus* genus with an average value of 63%. The next in order of increasing degree of divergence is the *Bavovirus* genus (71%). Both of these genera include avian caliciviruses.

**Figure 3:**
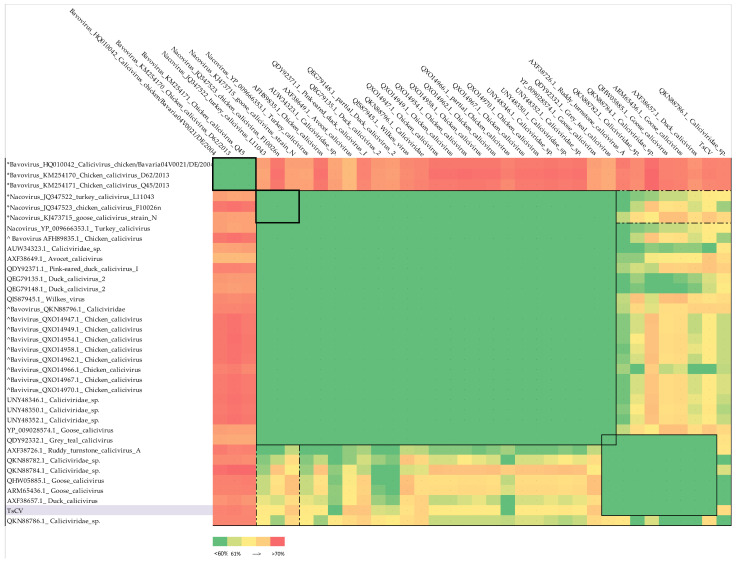
The pairwise divergence of the amino acid sequence of VP1, calculated for a set of sequences, including TsCV, representative sequences of the genera *Nacovirus* and *Bavovirus* (marked with *) and closest homologues of the TsCV VP1, presented as a heat map. The bright green color shows values of evolutionary distances less than 60%, which is the accepted criterion for genus demarcation. The color scale, from pale green to red, shows an increase in pairwise divergence from 60 to 73, the maximum value for the set under the study. Bold lines delimit areas of the map containing values for representative sequences of accepted genera. Thin lines separate clusters of values that match the criterion for a separate genus. The dotted line indicates the values used for comparison with the *Nacovirus* genus. The (^) denotes sequences presumably misclassified into the genus *Bavovirus*.

To establish the taxonomic relationship of TsCV with currently known caliciviruses with publicly available major capsid protein sequences, we performed a search for homologous proteins using the BLASTp algorithm and nr database [date of access: 15 May 2022]. List of top BLAST hits is shown in Table A3. This set consisted of unclassified caliciviruses, as well as representatives of the genera *Nacovirus* and *Bavovirus* according to the specified taxonomy. From each polyprotein sequence, a VP1 protein region was isolated based on sequence annotation and/or alignment with annotated members of the *Nacovirus* and *Bavovirus* genera (listed in the Table A2). Then, identical sequences were filtered out and representative sequences of *Bavovirus* and *Nacovirus* genera were added to the analyzed set. After that, the matrix of pairwise divergence of VP1 sequences was calculated, which is presented as a heat map (Figure 3).

Sequences belonging to unclassified caliciviruses are divided into two main clusters: a major one, which also includes representative sequences of *Nacovirus* genus, and a minor one, which consists entirely of unclassified caliciviruses, but includes the characterized goose calicivirus (KY399947.1, [6]). With this paper, the authors carried out a comparative and phylogenetic study showing that the discovered calicivirus did not belong to any of the accepted genera and proposed a new genus *Sanovirus*. Our results also support this assumption. Based on the given divergence values, considering the accepted criterion, it can be concluded that:

1. Goose calicivirus, (ARM65436.1 and QHW05885.1), duck calicivirus (AXF38657.1) and *Caliciviridae* sp. with accession numbers QKN88782.1, QKN88786.1 and QKN88784.1, as well as TsCV, cannot be assigned to the *Nacovirus* genus and can be combined into one separate genus with a member of the proposed *Sanovirus* genus included (the spread of divergence values within the proposed genus is 49.8 ± 7.8%, the divergence with members of the *Nacovirus* genus is 62.7 ± 1.7%).
2. Ruddy turnstone calicivirus A (AXF38726.1) has borderline divergence values from representatives of the genus *Nacovirus* (60.3 ± 0.8%) but is much closer to the proposed genus *Sanovirus* (values of pairwise divergence with goose calicivirus 53.3% and with putative members 56.1 ± 2.5%).
3. The inclusion of ruddy turnstone calicivirus A (AXF38726.1) virus in the proposed genus *Sanovirus* does not violate the demarcation criterion (the spread of divergence values within the proposed genus is 52.1 ± 7.2% and the divergence with members of the *Nacovirus* genus is 62.3 ± 1.8%).
4. Grey teal calicivirus (QDY92332.1) cannot be classified according to the accepted criterion, since the divergence values of its VP1 amino acid sequence are less than 60%, both in comparison with representative sequences of *Nacovirus* and with putative members of the proposed *Sanovirus* genus.
5. Chicken caliciviruses accession numbers QXO14947, QXO14949, QXO14954, QXO14958, QXO14962, QXO14966, QXO14967, QXO14970, AFH89835.1 and *Caliciviridae* sp. QKN88796 appear to be misclassified to the genus *Bavovirus* and should be moved to the genus *Nacovirus*.

### 3.3. Phylogenetic Analysis

A phylogenetic analysis was performed to determine the evolutionary relationship between TsCV and other *Caliciviridae* members. The tree was built on the basis of the amino acid sequences of major capsid proteins in accordance with the ICTV recommendations [4], since this sequence is the gold standard for identifying the *Caliciviridae* family.

**Figure 4:**
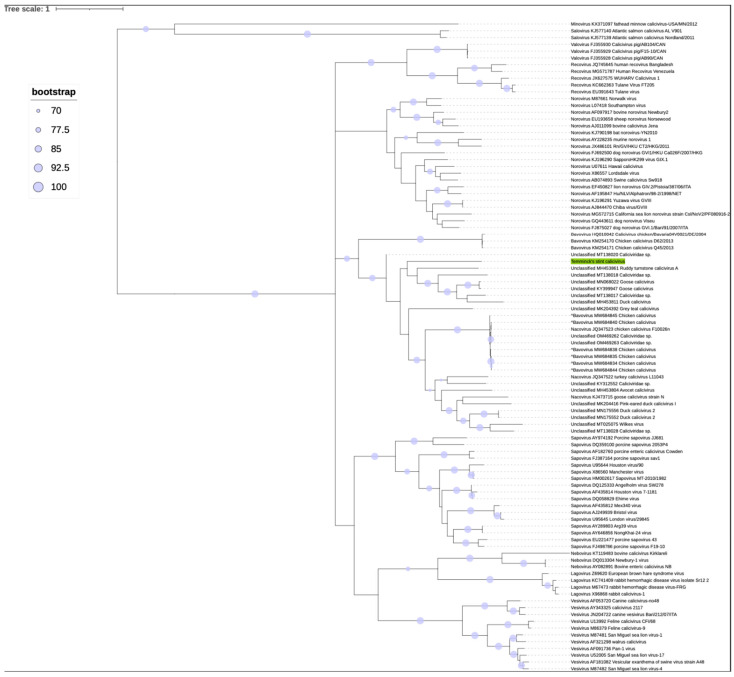
Phylogenetic tree constructed using the amino acid sequences of the VP1 protein of representative sequences of all accepted genera of the *Caliciviridae* family, TsCV (highlighted in green) and its closest relatives, found by analysis of VP1 protein homologues. The scale bar corresponds to the expected mean number of nucleotide substitutions per site. The support value of the TsCV branch is 58 (not shown). The (^) denotes sequences presumably misclassified into the genus *Bavovirus*; see Section 3.2.

The topology of external nodes of the obtained phylogenetic tree (Figure 4) was strongly supported by bootstrap values. The topology of the tree was consistent with the ICTV phylogeny that is traditionally used to characterize the *Caliciviridae* family [4]. Genome TsCV was located within the clade containing many unclassified members of the family, as well as members of the genera *Bavovirus* and *Nacovirus*. Since all of the characterized members of the clade have been isolated from birds, the clade appears to represent a group of related bird-infecting caliciviruses.

**Figure 5:**
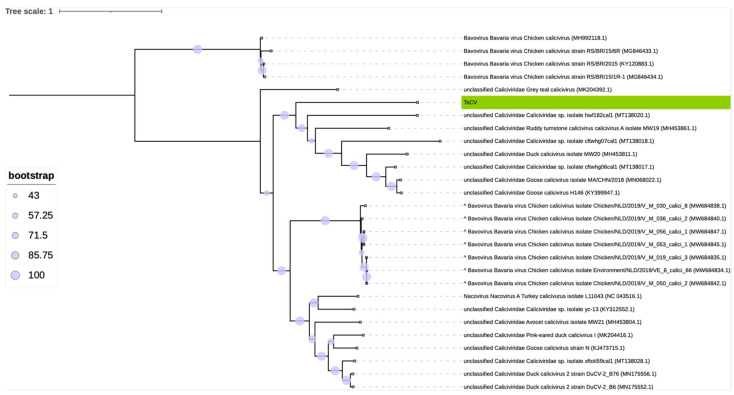
Phylogenetic tree constructed using the nucleotide sequences of the whole genome of representative sequences of all accepted genera of the *Caliciviridae* family, TsCV (highlighted in green) and its closest relatives, found by analysis of VP1 protein homologues. The scale bar corresponds to the expected mean number of nucleotide substitutions per site. The tree subdivides the genus *Bavovirus* into two non-closely related clades, resulting in the *Bavovirus* group not being monophyletic. The (^) denotes sequences presumably misclassified into the genus *Bavovirus*; see Section 3.2.

Since the topology in the clade of interest was not supported by high bootstrap values (the bootstrap value of the TsCV branch was 58), which can often be expected when constructing a phylogenetic tree of highly divergent sequences, we constructed a more accurate phylogenetic tree with a higher resolution on the basis of the whole genome nucleotide sequences of the TsCV and its closest relatives (Figure 5). The tree, regardless of the slight differences, is mostly consistent with the topology based on the amino acid sequences of the major capsid protein. Strong bootstrap support for TsCV clustering with other proposed *Sanovirus* sequences supports our hypothesis.

### 3.4. Species Demarcation

Various single-locus approaches based on the amino-acid sequence of the major capsid protein have been used to distinguish species. To create species partitions using paired patristic genetic distances, we employed the assemble species by automatic partitioning (ASAP) approach. The partition with the best ASAP score was selected. As a result of applying this method, the studied set of 96 *Caliciviridae* representatives was partitioned into 69 groups corresponding to different species. In addition, we applied the bPTP web interface, which uses the phylogenetic species concept to delimit species. Using both maximum likelihood and Bayesian approaches, 73 and 68 species partitions were identified, respectively. In both cases, the TsCV formed an independent operational taxonomic unit. Finally, using the GMYC method, 73 species groups with a single-threshold approach were identified (Table A4 contains complete data for the family *Caliciviridae*). 

The result shows that the TsCV genome is not partitioned with other genomes when using any of the listed models. Thus, we have shown the TsCV does not belong to a previously sequenced species of the *Caliciviridae* family. We propose assigning the TsCV virus to a new, previously undescribed species, preliminarily named Temminck’s stint calicivirus.

All three methods we used unanimously attributed the following groups of caliciviruses to common species:

1. Chicken calicivirus Q45/2013 (KM254171) and chicken calicivirus D62/2013 (KM254170) belonging the genus *Bavovirus*;
2. Bovine enteric calicivirus NB (AY082891) and Newbury-1 virus (DQ013304) belonging the genus *Nebovirus*;
3. Chiba virus/GVIII (AJ844470) and Yuzawa virus GVIII (KJ196291) belonging the genus *Norovirus*;
4. Calicivirus pig/AB104/CAN (FJ355930), calicivirus_pig/AB90/CAN (FJ355928) and calicivirus_pig/F15-10/CAN (FJ355929) belonging the genus *Valovirus*;
5. Sapovirus Angelholm virus SW278 (DQ125333), Ehime_virus (DQ058829) and Houston virus 7-1181 (AF435814) belonging the genus *Sapovirus*.
6. NongKhai-24 virus (AY646856) and Arg39 virus (AY289803) belonging the genus *Sapovirus*;
7. Sapovirus MT-2010/1982 (HM002617), sapovirus U65427 and Manchester virus (X86560) belonging the genus *Sapovirus*;
8. London_virus/29845 (U95645) and Bristol_virus (AJ249939) belonging the genus *Sapovirus*;
9. Rabbit_calicivirus-1 (X96868) and rabbit hemorrhagic disease virus-FRG (M67473) belonging the genus *Lagovirus*;
10. Unclassified duck calicivirus 2 MN175552 and MN175556;
11. Unclassified goose calicivirus KY399947 and MN068022;
12. Chicken calicivirus F10026n (JQ347523, *Nacovirus* according to the ICTV *Caliciviridae* report [4]), unclassified Caliciviridae_sp. OM469263 and OM469262 and six chicken caliciviruses strains (MW684845, MW684838, MW684835, MW684844, MW684834 and MW684840) presumably misclassified to the genus *Bavovirus*.

In addition, according to the species delimitation criteria used in this study, the following pairs of viruses should also be considered as the one species, since their major capsid protein sequences were exactly identical:

1. Goose calicivirus strain N (KJ473715) belonging the genus *Nacovirus* according to the ICTV *Caliciviridae* report [4] and ucclassified goose calicivirus (NC_024078);
2. *Caliciviridae sp.* OM469263.1 and OM469260.

### 3.5. Protein 3D-Structure Prediction

**Figure 6:**
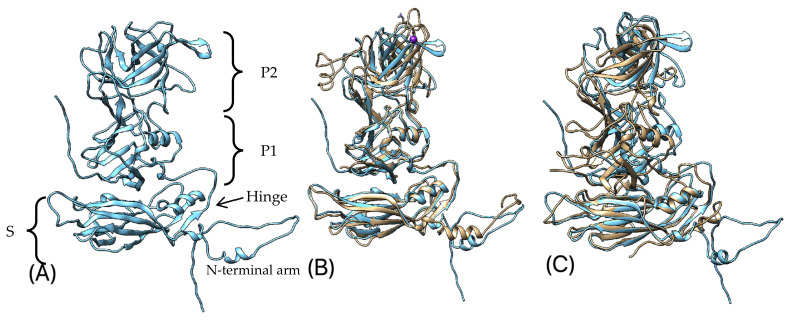
(**A**) Predicted 3D structure of VP1 protein of TsCV; (**B**)—predicted 3D structure of VP1 protein of TsCV (blue) aligned with VP1 protein of feline calicivirus strain F9, chain A (yellow) [17], a purple sphere represents the potassium ion—a ligand included in the crystal structure of the VP1 protein of feline calicivirus; (**C**)—predicted 3D structure of VP1 protein of TsCV (blue) aligned with VP1 protein of Norwalk virus (yellow) [70].

**Figure 7:**
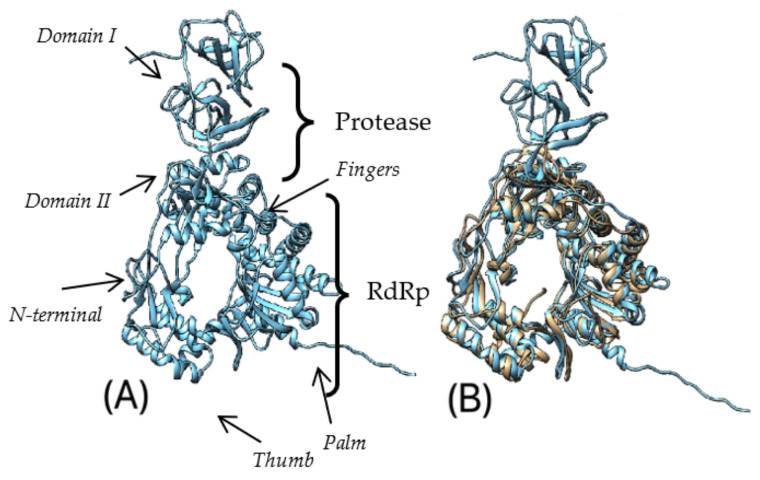

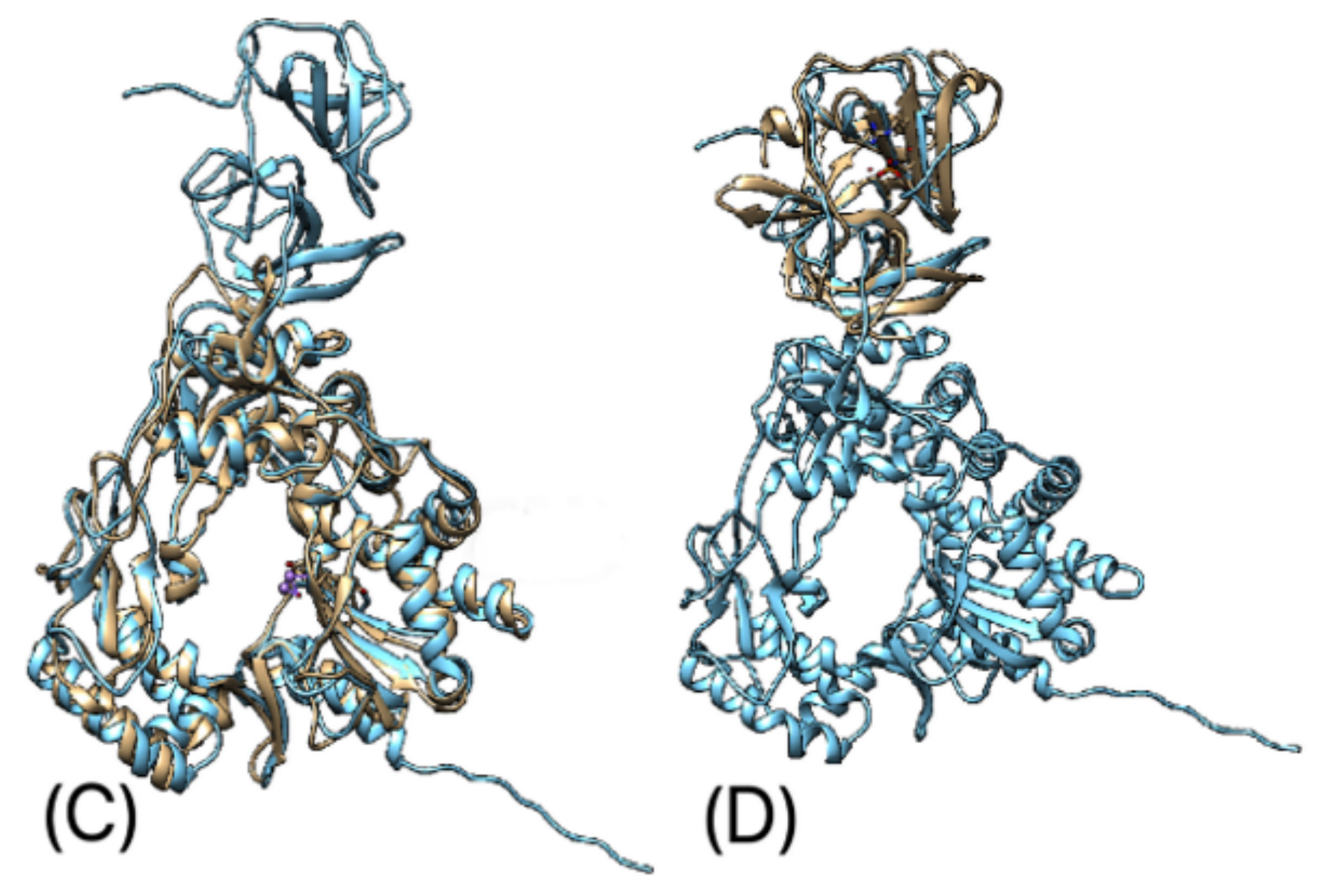
(**A**) Predicted 3D structure of Pro-Pol precursor protein of TsCV (domains of both pro-domains are written in italic); (**B**) predicted 3D structure of Pro-Pol precursor protein (blue) aligned with RdRp of *Norwalk virus* (yellow) [73]; (**C**) predicted 3D structure of Pro-Pol precursor protein (blue) aligned with RdRp of rabbit hemorrhagic disease virus (yellow) [72]; (**D**) predicted 3D structure of Pro-Pol precursor protein (blue) aligned with protease of *Norwalk virus* (yellow) [71].

**Figure 8:**
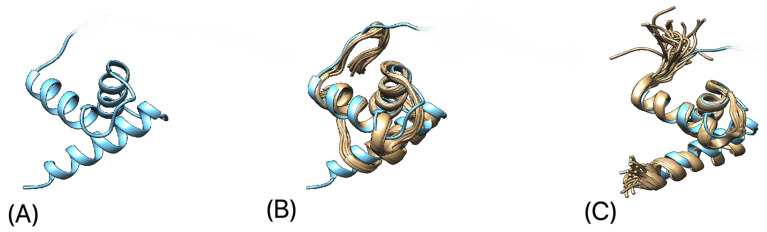
(**A**) Predicted 3D structure of VPg protein core domain of TsCV; (**B**) predicted 3D structure of VPg protein core domain of TsCV (blue) aligned with VPg protein core domain of feline calicivirus (yellow) [74]; (**C**) predicted 3D structure of VPg protein core domain of TsCV (blue) aligned with VPg protein core domain of porcine sapovirus (yellow) [75].

We predicted the 3D structure of the following TsCV proteins: the VP1 protein, the proteinase–polymerase precursor protein (Pro-Pol) and the core domain of the VPg protein. All predictions were made using the machine learning approach AlphaFold 2 [60] based on the primary sequence of each protein. Of the five models generated for each protein, the one with the highest per-residue confidence score (pLDDT) was selected (Figure 6A, Figure 7A, Figure 8A). 

The predicted VP1 protein has a structure typical of the major capsid protein of caliciviruses and consists of short N-terminal arm and two main domains, shell (S) and protruding (P), linked by a flexible hinge (Figure 6A). P domains divided into two subdomains, P1 and P2. S domain of TsCV VP1 has a classical for viral capsids structure of eight-stranded anti-parallel β sandwich with two well-defined α-helices and shows high structural similarity to other caliciviruses (on the example of known crystal structures of VP1 of feline calicivirus [17] and *Norwalk virus* [70]). The P1 domain, which shows a high correspondence of the spatial arrangement of structural elements with the feline calicivirus, differs from it by the presence of an additional short alpha helix and two extra β strands. The P2 domain, which includes the host-specific receptor binding site and major immunodominant epitopes [17], folds into β-barrel-like structure, but in comparison with Norwalk virus has two extra C-terminal β-strands.

The predicted structure of the Pro-Pol complex contains proteinase and polymerase pro-domains (Figure 7A). Domain I of TsCV structurally resembles *Norwalk virus* [71], but misses two α-helices: one at the C-terminus end and in the proximal part of the domain. Domain II folds into a β-barrel-like structure, similar to *Norwalk virus*, but misses a β-strand and a short α-helix in the proximal part of the domain. The polymerase pro-domain shows high structural similarity to that of both rabbit hemorrhagic disease virus (RHDV) [72] and *Norwalk virus* [73], typical for three-dimensional structures of most other polynucleotide polymerases. The N-terminal region of TsCV contains two additional short β-strands compared to *Norwalk virus* (or one, compared to RHDV) and misses a short α-helix present in RHDV. Several additional short β-strands compared to both RHDV and *Norwalk virus* are also found in the fingers domain. An additional α-helix in the C-terminal part of the thumb domain was predicted in the TsCV polymerase pro-domain.

The core domain of the predicted 3D structure of VPg protein adopts a helical structure with N-terminus and C-terminus regions at two separate ends of the domain. The structure of the TsCV VPg core domain is highly similar to feline calicivirus [74] and porcine sapovirus [75] VPg.

For the VP2 protein of TsCV, we were unable to obtain a 3D structure with acceptable pLDDT scores (the highest pLDDT score was 43.09); however, according to all the models obtained, the core part of the VP2 protein of TsCV consists of three consecutive alpha-helices—one long and two short. The predicted order of the secondary structures was in complete agreement with the structure of the VP2 protein of the feline calicivirus obtained with cryo-electron microscopy [17].

## 4. Discussion

With the development of metagenomics, the inability to obtain a culture of microorganisms has ceased to be a problem that limits the ability to characterize the species diversity of an ecological niche and study the genomic features of its representatives. Here, we identified and characterized the complete genome of a novel Temminck’s stint calicivirus (TsCV), isolated from the wild bird *Calidris temminckii* captured in Russia, Siberia. Using metagenomic data, we were able to obtain the complete genome sequence of TsCV, annotate its CDS, describe its proteins and model their 3D structure, and carry out taxonomic classification and phylogenetic study. 

We showed that the structure of the TsCV genome corresponds to that of the accepted genera *Lagovirus, Nacovirus, Nebovirus, Sapovirus* and *Valovirus*, for which the coding part is organized into two open reading frames encoding the polyprotein and VP1. The TsCV polyprotein has all the expected proteins, arranged in an order conservative among all caliciviruses [76]. However, TsCV is genetically distant from all known caliciviruses and, in addition, has a different relative arrangement of open reading frames, indicating that this virus apparently does not use a termination-re-initiation mechanism during VP2 translation [77,78], as suggested for all caliciviruses [4].

The spatial similarity of the predicted 3D structures of TsCV proteins and the crystal structures of their homologues shows that machine-learning approaches can be successfully applied to model the caliciviral proteins and additionally confirms the CDS annotation. The ability to obtain the 3D structure of the caliciviral VP1 protein is a task of particular importance, since in caliciviruses, capsid-related functions, such as antigenicity and host specificity, are predominantly determined by its primary sequence and spatial configuration [79].

Metagenomic sequencing has provided researchers with a multitude of genomes, many of which require de novo classification. Development and improvement of bioinformatics methods and robust classification of metagenomic sequences allows a significant expansion of the formal taxonomy of viruses in the way of future studies of virus diversity [80,81]. However, it is obvious that by using this approach, most of the recommended species and genus classification criteria are inaccessible to the researcher. However, on the other hand, most of the traditional species-defining traits [82] have not been fully characterized for most caliciviruses since all of them require viral cultures [1]. Therefore, species-delimitation methods based mainly on genomic information are gaining popularity. Such methods include ANI (average nucleotide identity) and ANI-like approaches based on pairwise distances between genome nucleotide sequences [83], as well as single-locus distances methods. In the case of caliciviruses, a recognized criterion for the delimitation of genera is the percentage identity of the amino acid sequence of the major capsid protein [4], while there is no single criterion for the division of species at all. Thus, such methods, although widely used, exhibit disadvantages, among which is the need to a priori establish a threshold for taxonomic delimitation, which in some cases cannot be correctly established. The taxonomic classification of TsCV according to the criteria established by the international committee does not allow it to be placed in one of the accepted genera of the *Caliciviridae* family. Then we applied the ICTV genus delimitation criterion to the unclassified viruses belonging to the *Caliciviridae* family and showed that TsCV can be assigned to the proposed *Sanovirus* genus [6] together with goose calicivirus, (KY399947 [6] and MN068022), duck calicivirus (MH453811, [67]) and *Caliciviridae* sp. with accession numbers MT138017, MT138020 and MT138018.

The general definition developed by the *Caliciviridae* Study Group (CSG) for a caliciviral species was as follows: “A calicivirus species will be defined as a cluster of viruses that constitutes a major phylogenetic branch within a genus and is also distinguishable from other branch(es) by one or more of the following biologic properties: natural host range, natural cell and tissue tropism, and antigenicity” [83]. As can be seen from the obtained phylogenetic trees (Figure 4 and Figure 5), the clade containing TsCV, which we propose to consider as a separate genus, is characterized by long branches (except for goose caliciviruses KY399947 [6] and MN068022). According to this criterion, there is no reason to believe that the TsCV virus forms a species together with other closely related published caliciviruses.

We tested this assumption using several other single-locus methods for species delimitation. In this study, to classify species, we used GMYC and bPTP coalescent-based methods that combine population genetic and phylogenetic theory to provide an objective means for the delimitation of evolutionarily significant units of diversity [53,54]. In addition to the methodologies mentioned above, the ASAP method was applied in this research. Compared to GMYC and PTP, ASAP utilizes a phenetic approach where similar sequences are clustered in the same group/species [55]. Since the tools use different approaches, combining them improves the accuracy of the analysis. The most reliable can be considered species partitions, confirmed by several different methods [45]. The approaches we apply have the benefit of proposing de novo species divisions and requiring no a priori-defined intraspecific genetic distances. All the methods we used classified the virus TsCV as a separate novel species. All the methods also confirmed that the remaining members of this proposed genus also belong to separate species, except for goose caliciviruses KY399947 [6] and MN068022, which appear to be strains of the same species. In addition, these methods have revealed groups of caliciviruses that are very closely related and could potentially be considered as strains of the same species.

Despite the ICTV approach being traditional and widely used, we applied an additional method to obtain a more accurate phylogenetic tree with a higher resolution. For this, we applied the approach described in [52] with the only difference being that we used whole genome sequences to construct an alignment. Since the genome of the TsCV is extremely divergent from all other known representatives of the family, we used sequences belonging only to the closest relatives of the TsCV virus. This allowed us to obtain a reliable phylogenetic tree containing the representatives of *Bavovirus* and *Nacovirus* genera, as well as unclassified caliciviruses, among which we localized the new TsCV virus. The tree demonstrates some minor differences in topology, e.g., the localization of branches MK204392.1 and MT138020.1. It should be noted that the nucleotide-based topologies are based on more phylogenetic information than amino acid-based topologies. In addition, it was shown that the use of alignment-editing methods allows the obtaining of a more correct topology, although sometimes with less robust supports [46].

Several viral species known to generate major disease burdens in people and animals, such as influenza viruses, West Nile virus, and Newcastle disease virus, have natural reservoirs in birds. Migratory birds, as a result, play a crucial role in the development and spread of dangerous viruses [58]. Extensive metagenomic investigations have significantly increased our understanding of the viromes of various ecosystems in recent years, including the identification of new viruses in domestic and wild bird species [84]. Caliciviruses have previously been detected in migratory birds such as ruddy turnstones (*Arenaria interpres*) [18]. To our knowledge, Temminck’s stint has not previously been described as a host for caliciviruses.

Temminck’s stint breeds in the north of Eurasia, mainly from Scandinavia to the east to Chukotka, Anadyr and Kamchatka, with more than 93% of the population occurring in Russia [85,86]. A typical migratory bird, it winters in the tropical climates of southern Europe, Africa and South and Southeast Asia. Infections transmitted by migrating birds potentially have the ability to travel long distances. Therefore, the Temminck’s stint can be a source of viral spreading in the Russia and other countries. Migratory bird virome characterization can help monitor potential infectious disease outbreaks in poultry and other animals, including humans.

## Figures and Tables

**Table 1:** Comparison the nucleotide sequence and structure of TeAdV-1 genome with the genomes of the closest relatives.

	TsCV	*Ruddy turnstone calicivirus A* [67] (MH453861.1)	Duck Calicivirus [67] (MH453811.1)	Goose Calicivirus (MN068022.1)	Goose Calicivirus [6] (KY399947.1)	*Caliciviridae* sp. (MT138017.1)	*Caliciviridae* sp. (MT138020.1)
%GC	51.73%	51.77%	50.71%	48.66%	49.13%	47.93%	50.78%
Genome nucleotide identity to TsCV		43.68%	40.33%	42.39%	41.82%	42.64%	42.37%
VP1 protein divergence with TsCV		59.70%	58.42%	57.44%	57.44%	59.69%	59.18%
3′ UTR length	816 nt	521 nt	901 nt	15 nt	18 nt	225 nt	731 nt
ORF1 length	6849 nt	7221 nt	7827 nt	7254 nt	7254 nt	7827 nt	7173 nt
Distance between ORF1 and ORF2	1 nt	−17 nt	−74 nt	−8 nt	−8 nt	−8 nt	−10 nt
ORF2 length	711 nt	621 nt	765 nt	855 nt	855 nt	852 nt	957 nt
5′ URT length	198 nt	452 nt	289 nt	323 nt	330 nt	99 nt	92 nt
Genome length	8575 nt	8798 nt	9780 nt	8439 nt	8449 nt	8995 nt	8943 nt

**Table 2:** Pairwise divergence by the VP1 protein sequence of ICTV representatives for *Caliciviridae* family and TsCV. The numbers indicate the average values of the specified parameter and its standard deviation within the compared taxonomic groups. The standard deviation calculation method is not applicable to the *Minovirus* genus, since only one sequence is available.

	*Bavovirus*	*Lagovirus*	*Minovirus*	*Nacovirus*	*Nebovirus*	*Norovirus*	*Recovirus*	*Salovirus*	*Sapovirus*	*Valovirus*	*Vesivirus*
**TsCV**	70.8 ± 0.3%	78.4 ± 0.3%	87.7	63.1 ± 1.7%	78.1 ± 0.3%	82.0 ± 1.4%	83.7 ± 0.2%	87.4 ± 0.9%	71.2 ± 1.2%	82.7 ± 0.3%	75.1 ± 1.1%

## Data Availability

Temminck’s stint calicivirus complete genome and annotation available in the GenBank database, accession number ON815296.

## Appendix A

**Table A1:** List of contigs assigned to eukaryotes. Statistics are given for contigs with a length of more than 600 nucleotides and taxonomic groups with a total length of contigs of more than 2000 nucleotides.

Taxa	Number of Contigs	Total Length of Contigs	Group
*Scolopacidae/Calidris*	21	19,912	Bird
*Turdidae/Erithacus*	2	2730	
*Sylviidae/Curruca*	3	2430	
*Amphipleuraceae/Halamphora*	3	3460	Algae
*Bacillariaceae/Cylindrotheca*	3	2757	
*Stauroneidaceae/Stauroneis*	3	2574	
*Danionidae/Danio*	6	7526	Fish
*Serranidae/Epinephelus*	4	3906	
*Batrachoididae/Thalassophryne*	2	2494	
*Apogonidae/Sphaeramia*	3	2442	
*Bovichtidae/Cottoperca*	2	2420	
*Echeneidae/Echeneis*	2	2005	
*Acanthosomatidae/Elasmucha*	5	5581	Insect
*Chironomidae/Chironomus*	4	4822	
*Apidae/Apis*	2	2064	
*Ostreidae/Crassostrea*	5	6755	Mollusc
*Eimeriidae/Eimeria*	7	7273	Parasite
*Rhabditidae/Caenorhabditis*	2	3541	
*Cornaceae/Cornus*	8	7911	Plant
*Malvaceae/Gossypium*	4	4437	
*Solanaceae/Solanum*	4	4272	
*Poaceae/Panicum*	2	2716	
*Brassicaceae/Brassica*	3	2585	
*Fabaceae/Cercis*	1	2406	
*Fabaceae/Cicer*	1	2348	
*Hominidae/Homo*	4	4027	Human
*Cercopithecidae/Macaca*	1	2868	Primate
*Amoebidiaceae/Amoebidium*	4	3011	Protozoa
*Protaspidae/Cryothecomonas*	1	2136	
*Octodontidae/Octodon*	1	2964	Rodent
*Sciuridae/Sciurus*	2	2732	
*Vespertilionidae/Eptesicus*	1	2138	Bat

**Table A2:** List of protein sequences used for comparative and phylogenetic analysis. A set of representative sequences accepted by ICTV.

Genus	Sequence Name	GenBank Accession
*Bavovirus*	calicivirus chicken/Bavaria04V0021/DE/2004	HQ010042
*Bavovirus*	chicken calicivirus D62/2013	KM254170
*Bavovirus*	chicken calicivirus Q45/2013	KM254171
*Lagovirus*	rabbit hemorrhagic disease virus isolate Sr12 2	KC741409
*Lagovirus*	rabbit hemorrhagic disease virus-FRG	M67473
*Lagovirus*	rabbit calicivirus-1	X96868
*Lagovirus*	European brown hare syndrome virus	Z69620
*Minovirus*	fathead minnow calicivirus-USA/MN/2012	KX371097
*Nacovirus*	turkey calicivirus L11043	JQ347522
*Nacovirus*	chicken calicivirus F10026n	JQ347523
*Nacovirus*	goose calicivirus strain N	KJ473715
*Nebovirus*	Newbury-1 virus	DQ013304
*Nebovirus*	bovine enteric calicivirus NB	AY082891
*Nebovirus*	bovine calicivirus Kirklareli	KT119483
*Norovirus*	lion norovirus GIV.2/Pistoia/387/06/ITA	EF450827
*Norovirus*	swine calicivirus Sw918	AB074893
*Norovirus*	bovine norovirus Newbury2	AF097917
*Norovirus*	Hu/NLV/Alphatron/98-2/1998/NET	AF195847
*Norovirus*	bovine calicivirus Jena	AJ011099
*Norovirus*	Chiba virus/GVIII	AJ844470
*Norovirus*	murine norovirus 1	AY228235
*Norovirus*	sheep norovirus Norsewood	EU193658
*Norovirus*	dog norovirus GVI1/HKU Ca026F/2007/HKG	FJ692500
*Norovirus*	dog norovirus GVI.1/Bari/91/2007/ITA	FJ875027
*Norovirus*	dog norovirus Viseu	GQ443611
*Norovirus*	Rn/GV/HKU CT2/HKG/2011	JX486101
*Norovirus*	Yuzawa virus GVIII	KJ196291
*Norovirus*	bat norovirus-YN2010	KJ790198
*Norovirus*	Southampton virus	L07418
*Norovirus*	Norwalk virus	M87661
*Norovirus*	California sea lion norovirus strain Csl/NoV2/PF080916-2	MG572715
*Norovirus*	Hawaii calicivirus	U07611
*Norovirus*	SapporoHK299 virus GIX.1	KJ196290
*Norovirus*	Lordsdale virus	X86557
*Recovirus*	Tulane virus	EU391643
*Recovirus*	human recovirus Bangladesh	JQ745645
*Recovirus*	human recovirus Venezuela	MG571787
*Recovirus*	WUHARV Calicivirus 1	JX627575
*Recovirus*	Tulane virus FT205	KC662363
*Salovirus*	Atlantic salmon calicivirus Nordland/2011	KJ577139
*Salovirus*	Atlantic salmon calicivirus AL V901	KJ577140
*Sapovirus*	porcine enteric calicivirus Cowden	AF182760
*Sapovirus*	Mex340 virus	AF435812
*Sapovirus*	Houston virus 7-1181	AF435814
*Sapovirus*	Arg39 virus	AY289803
*Sapovirus*	NongKhai-24 virus	AY646856
*Sapovirus*	porcine sapovirus JJ681	AY974192
*Sapovirus*	Ehime virus	DQ058829
*Sapovirus*	Angelholm virus SW278	DQ125333
*Sapovirus*	porcine sapovirus 2053P4	DQ359100
*Sapovirus*	porcine sapovirus 43	EU221477
*Sapovirus*	porcine sapovirus sav1	FJ387164
*Sapovirus*	porcine sapovirus F19-10	FJ498786
*Sapovirus*	Sapovirus MT-2010/1982	HM002617
*Sapovirus*	Sapporo virus	U65427
*Sapovirus*	Houston virus/90	U95644
*Sapovirus*	Manchester virus	X86560
*Sapovirus*	Bristol virus	AJ249939
*Sapovirus*	London virus/29845	U95645
*Valovirus*	calicivirus pig/AB90/CAN	FJ355928
*Valovirus*	calicivirus pig/F15-10/CAN	FJ355929
*Valovirus*	calicivirus pig/AB104/CAN	FJ355930
*Vesivirus*	canine calicivirus-no48	AF053720
*Vesivirus*	Pan-1 virus	AF091736
*Vesivirus*	vesicular exanthema of swine virus strain A48	AF181082
*Vesivirus*	walrus calicivirus	AF321298
*Vesivirus*	calicivirus 2117	AY343325
*Vesivirus*	canine vesivirus Bari/212/07/ITA	JN204722
*Vesivirus*	Feline calicivirus-9	M86379
*Vesivirus*	San Miguel sea lion virus-1	M87481
*Vesivirus*	San Miguel sea lion virus-4	M87482
*Vesivirus*	feline calicivirus CFI/68	U13992
*Vesivirus*	San Miguel sea lion virus-17	U52005

**Table A3:** List of top BLAST hits obtained by analyzing the VP1 amino acid sequence of TsCV using the BLASTp search algorithm and the nr database of the NCBI.

GenBank Accession	Sequence Name
QKN88784.1	*Caliciviridae sp.*
QHW05885.1	Goose calicivirus
AXF38657.1	Duck calicivirus
ARM65436.1	Goose calicivirus
QKN88782.1	*Caliciviridae sp.*
AXF38726.1	*Ruddy turnstone calicivirus A*
AUW34323.1	*Caliciviridae sp.*
QKN88786.1	*Caliciviridae sp.*
QDY92332.1	Grey teal calicivirus
QKN88796.1	*Caliciviridae sp.*
YP_9666353.1	Turkey calicivirus
QIS87945.1	Wilkes virus
AFH89835.1	Chicken calicivirus
QEG79135.1	Duck calicivirus 2
QEG79148.1	Duck calicivirus 2
QDY92371.1	Pink-eared duck calicivirus I
QXO14949.1	Chicken calicivirus
QXO14947.1	Chicken calicivirus
QXO14962.1	Chicken calicivirus
QXO14958.1	Chicken calicivirus
UNY48352.1	*Caliciviridae sp.*
UNY48346.1	*Caliciviridae sp.*
QXO14954.1	Chicken calicivirus
UNY48350.1	*Caliciviridae sp.*
QXO14967.1	Chicken calicivirus
YP_9028574.1	Goose calicivirus
AXF38649.1	Avocet calicivirus
QXO14966.1	Chicken calicivirus
QXO14970.1	Chicken calicivirus

**Table A4:** Species delimitation schemes were obtained using the ASAP, PTP and GMYC approaches. The values in the cells correspond to the number of representatives per taxonomic unit.

Virus	ASAP	bPTP ML	bPTP Bayesian	GMYC (Single Threshold)
Minovirus_KX371097_fathead_minnow_calicivirus-USA/MN/2012	1	1	1	outgroup
Salovirus_KJ577139_Atlantic_salmon_calicivirus_Nordland/2011	1	1	1	outgroup
Salovirus_KJ577140_Atlantic_salmon_calicivirus_AL_V901	1	1	1	outgroup
Bavovirus_HQ010042_Calicivirus_chicken/Bavaria04V0021/DE/2004	3	3	3	2
Bavovirus_KM254171_Chicken_calicivirus_Q45/2013				
Bavovirus_KM254170_Chicken_calicivirus_D62/2013				1
Temminck’s stint calicivirus	1	1	1	1
Unclassified_MT138020_Caliciviridae_sp.	1	1	1	1
Nebovirus_KT119483_bovine_calicivirus_Kirklareli	1	1	1	1
Nebovirus_AY082891_Bovine_enteric_calicivirus_NB	2	2	2	2
Nebovirus_DQ013304_Newbury-1_virus				
^Bavovirus_MW684845_Chicken_calicivirus	9	9	9	9
^Bavovirus_MW684838_Chicken_calicivirus				
^Bavovirus_MW684835_Chicken_calicivirus				
^Bavovirus_MW684844_partial_Chicken_calicivirus				
^Bavovirus_MW684834_Chicken_calicivirus				
^Bavovirus_MW684840_Chicken_calicivirus				
Nacovirus_JQ347523_chicken_calicivirus_F10026n				
Unclassified_OM469263_Caliciviridae_sp.				
Unclassified_OM469262_Caliciviridae_sp.				
Unclassified_MK204392_Grey_teal_calicivirus	1	1	1	1
Unclassified_MH453861_Ruddy_turnstone_calicivirus_A	1	1	1	1
Valovirus_FJ355930_Calicivirus_pig/AB104/CAN	3	3	3	3
Valovirus_FJ355928_Calicivirus_pig/AB90/CAN				
Valovirus_FJ355929_Calicivirus_pig/F15-10/CAN				
Unclassified_MH453804_Avocet_calicivirus	1	1	1	1
Unclassified_MT138018_Caliciviridae_sp.	1	1	1	1
Unclassified_MK204416_Pink-eared_duck_calicivirus_I	1	1	1	1
Nacovirus_KJ473715_goose_calicivirus_strain_N	1	1	1	1
Sapovirus_DQ125333_Angelholm_virus_SW278	3	3	3	3
Sapovirus_DQ058829_Ehime_virus				
Sapovirus_AF435814_Houston_virus_7-1181				
Norovirus_KJ790198_bat_norovirus-YN2010	1	1	1	1
Norovirus_FJ692500_dog_norovirus_GVI1/HKU_Ca026F/2007/HKG	1	1	1	1
Unclassified_KY312552_Caliciviridae_sp.	1	1	1	1
Nacovirus_JQ347522_turkey_calicivirus_L11043	1	1	1	1
Unclassified_MH453811_Duck_calicivirus	1	1	1	1
Unclassified_MT138017_Caliciviridae_sp.	1	1	1	1
Unclassified_KY399947_Goose_calicivirus	2	2	2	2
Unclassified_MN068022_Goose_calicivirus				
Norovirus_MG572715_California_sea_lion_norovirus_strain_Csl/NoV2/PF080916-2	1	1	1	1
Norovirus_AJ844470_Chiba_virus/GVIII	2	2	2	2
Norovirus_KJ196291_Yuzawa_virus_GVIII				
Sapovirus_DQ359100_porcine_sapovirus_2053P4	1	1	1	1
Sapovirus_AY974192_Porcine_sapovirus_JJ681	1	1	1	1
Unclassified_MT138028_Caliciviridae	1	1	1	1
Unclassified_MT025075_Wilkes_virus	1	1	1	1
Unclassified_MN175552_Duck_calicivirus_2	2	2	2	2
Unclassified_MN175556_partial_Duck_calicivirus_2				
Sapovirus_AY646856_NongKhai-24_virus	2	2	2	2
Sapovirus_AY289803_Arg39_virus				
Norovirus_KJ196290_SapporoHK299_virus_GIX.1	1	1	1	1
Norovirus_FJ875027_dog_norovirus_GVI.1/Bari/91/2007/ITA	1	1	1	1
Norovirus_GQ443611_dog_norovirus_Viseu	1	1	1	1
Norovirus_U07611_Hawaii_calicivirus	1	1	1	1
Norovirus_JX486101_Rn/GV/HKU_CT2/HKG/2011	1	1	1	1
Norovirus_AY228235_murine_norovirus_1	1	1	1	1
Norovirus_AB074893_Swine_calicivirus_Sw918	1	1	1	1
Norovirus_X86557_Lordsdale_virus	1	1	1	1
Norovirus_AF195847_Hu/NLV/Alphatron/98-2/1998/NET	1	1	1	1
Norovirus_EF450827_lion_norovirus_GIV.2/Pistoia/387/06/ITA	1	1	1	1
Norovirus_L07418_Southampton_virus	1	1	1	1
Norovirus_M87661_Norwalk_virus	1	1	1	1
Vesivirus_AF053720_Canine_calicivirus-no48	1	1	1	1
Recovirus_MG571787_Human_Recovirus_Venezuela	1	1	1	1
Recovirus_JQ745645_human_recovirus_Bangladesh	1	1	1	1
Norovirus_AF097917_bovine_norovirus_Newbury2	1	1	1	1
Norovirus_AJ011099_bovine_calicivirus_Jena	1	1	1	1
Norovirus_EU193658_sheep_norovirus_Norsewood	1	1	1	1
Sapovirus_U95644_Houston_virus/90	1	1	1	1
Sapovirus_HM002617_Sapovirus_MT-2010/1982	2	2	2	2
Sapovirus_X86560_Manchester_virus				
Vesivirus_AF091736_Pan-1_virus	1	1	4	1
Vesivirus_U52005_San_Miguel_sea_lion_virus-17	1	1		1
Vesivirus_M87482_San_Miguel_sea_lion_virus-4	2	1		1
Vesivirus_AF181082_Vesicular_exanthema_of_swine_virus_strain_A48		1		1
Lagovirus_Z69620_European_brown_hare_syndrome_virus	1	1	1	1
Lagovirus_KC741409_rabbit_hemorrhagic_disease_virus_isolate_Sr12_2	3	3	3	1
Lagovirus_X96868_rabbit_calicivirus-1				2
Lagovirus_M67473_rabbit_hemorrhagic_disease_virus-FRG				
Sapovirus_FJ498786_porcine_sapovirus_F19-10	1	1	1	1
Sapovirus_EU221477_porcine_sapovirus_43	1	1	1	1
Recovirus_JX627575_WUHARV_Calicivirus_1	1	1	1	1
Recovirus_EU391643_Tulane_virus	2	2	2	1
Recovirus_KC662363_Tulane_Virus_FT205				1
Vesivirus_JN204722_canine_vesivirus_Bari/212/07/ITA	2	1	1	1
Vesivirus_AY343325_calicivirus_2117		1	1	1
Vesivirus_AF321298_walrus_calicivirus	1	1	1	1
Vesivirus_M87481_San_Miguel_sea_lion_virus-1	1	1	1	1
Sapovirus_AF435812_Mex340_virus	1	1	1	1
Sapovirus_U95645_London_virus/29845	2	2	2	2
Sapovirus_AJ249939_Bristol_virus				
Sapovirus_FJ387164_porcine_sapovirus_sav1	2	1	2	1
Sapovirus_AF182760_porcine_enteric_calicivirus_Cowden		1		1
Vesivirus_M86379_Feline_calicivirus-9	2	1	2	1
Vesivirus_U13992_Feline_calicivirus_CFI/68		1		1
TOTAL:	68	72	67	72
